# Fabrication and Characterization of Flexible Thermoelectric Generators Using Micromachining and Electroplating Techniques

**DOI:** 10.3390/mi10100660

**Published:** 2019-09-30

**Authors:** Wnag-Lin Lee, Po-Jen Shih, Cheng-Chih Hsu, Ching-Liang Dai

**Affiliations:** 1 Department of Mechanical Engineering, National Chung Hsing University, Taichung 402, Taiwan; victor20080610@hotmail.com; 2 Department of Biomedical Engineering, National Taiwan University, Taipei 106, Taiwan; pjshih@ntu.edu.tw; 3 Department of Electro-Optical Engineering, National United University, Miaoli 360, Taiwan; cchsu920624@nuu.edu.tw

**Keywords:** thermoelectric generator, flexibility, micromachining, electroplating, thermocouple

## Abstract

This study involves the fabrication and measurement of a flexible thermoelectric generator (FTG) using micromachining and electroplating processes. The area of the FTG is 46 × 17 mm^2^, and it is composed of 39 thermocouples in series. The thermoelectric materials that are used for the FTG are copper and nickel. The fabrication process involves patterning a silver seed layer on the polymethyl methacrylate (PMMA) substrate using a computer numerical control (CNC) micro-milling machine. Thermoelectric materials, copper and nickel, are deposited on the PMMA substrate using an electroplating process. An epoxy polymer is then coated onto the PMMA substrate. Acetone solution is then used to etch the PMMA substrate and to transfer the thermocouples to the flexible epoxy film. The FTG generates an output voltage (OV) as the thermocouples have a temperature difference (Δ*T*) between the cold and hot parts. The experiments show that the OV of the FTG is 4.2 mV at Δ*T* of 5.3 K and the output power is 429 nW at Δ*T* of 5.3 K. The FTG has a voltage factor of 1 μV/mm^2^K and a power factor of 19.5 pW/mm^2^K^2^. The FTG reaches a curvature of 20 m^−1^.

## 1. Introduction

Many recent studies have focused on the development of thermoelectric generators [1,2,3,4,5,6,7,8]. Thermoelectric generators are used to convert waste heat into electrical power, which is a green energy. It is difficult to use a non-flexible thermoelectric generator for a heat source with a curved surface because it does not fit the curved surface. However, a flexible thermoelectric generator can be used for a heat source with a curved surface and has more applications than a non-flexible thermoelectric generator. Flexible thermoelectric generators can be used as a wearable device to fit the curves of human skin.

Various fabrication methods were used for thermoelectric generators. For instance, Lee [9] used a screen printing method to produce a thermoelectric generator using the thermoelectric materials, ZnSb and CoSb_3_, on an alumina substrate. The output voltage (OV) for the thermoelectric generator was 10 mV at Δ*T* of 50 K, and its output power per unit area was 1.7 μW/mm^2^ at temperature difference (Δ*T*) of 50 K. The power factor of the thermoelectric generator was 0.68 pW/mm^2^K^2^. Phaga [10] developed a thermoelectric generator that had 31 thermocouples in series. The thermocouple materials were p-Ca_3_Co_4_O_9_ and n-CaMnO_3_ that were deposited by the solid state reaction method. The area of the thermoelectric generator was 6.45 cm^2^. The OV for the thermoelectric generator was 121.7 mV at Δ*T* of 140 K and the output power was 1.47 μW at Δ*T* of 140 K. The voltage factor for the thermoelectric generator was 1.35 μV/mm^2^K and the power factor was 0.16 pW/mm^2^K^2^. Big-Alabo [11] produced a thermoelectric generator using low pressure chemical vapor deposition and a micro-fabrication process [12]. Ge and SiGe were the thermoelectric materials. The thermoelectric generator had an estimated power density of 111 nW/cm^2^ and a power factor of 0.0035 μW/cm^2^K^2^. Itoigawa [13] proposed a flexible thermoelectric generator that was fabricated on a polyimide substrate using a micro-fabrication process. The thermoelectric materials for the generator were copper and nickel. The generator contained 380 thermocouples in series, and its area was about 50 × 50 mm^2^. The generator had a bending radius of curvature of 9 mm. The OV and output power were 16 μV/K per thermocouple and 4.1 pW/K^2^ per thermocouple, respectively. Therefore, the thermoelectric generator had a voltage factor of 2.43 μV/mm^2^K and a power factor of 0.64 pW/mm^2^K^2^. A flexible thermoelectric generator, developed by Lu [14], was fabricated using physical mixing and drop casting processes. The thermoelectric materials for the generator were Te/poly(3,4-ethylenedioxythiophene) and poly(styrenesulfonate) /Cu_7_Te_4_ ternary composite films. The flexible thermoelectric generator (FTG) consisted of eight thermopiles with Ag paste and the area was about 25 × 40 mm^2^. The OV for the generator was 31.2 mV at Δ*T* of 39 K and the maximum output power was 94.7 nW at Δ*T* of 39 K. The FTG had a power factor of 112.3 μW/mK^2^. Ding [15] manufactured a flexible thermoelectric generator on a nylon membrane. The thermoelectric material of the generator was n-type Ag_2_Se. The FTG contained four legs of the Ag_2_Se film that were connected with Ag paste on a nylon substrate and the area of the FTG was about 20 × 20 mm^2^. The OV and the maximum output power for the FTG were 18 mV and 460 nW, respectively, at Δ*T* of 30 K. The voltage factor, the FTG was 1.5 μV/mm^2^K and the power factor was 1.2 pW/mm^2^K^2^. Selvan [16] manufactured a thermoelectric generator on a polyimide substrate using a microfabrication process. Cobalt and copper were used as negative and positive thermoelectric materials for the thermoelectric generator. The generator was a flexible sandwiched planar structure. The power factor for the thermoelectric generator was 6.6 × 10^−3^ μW/cm^2^K^2^ at Δ*T* of 44.2 K. A flexible thermoelectric generator, presented by Jo [17], was made on a polydimethylsiloxane substrate to harvest heat energy from a human body. The materials for the thermocouples in the generator were n-type and p-type Bi_2_Te_3_, which were manufactured using dispenser printing. The area of the thermoelectric generator was 50 × 50 mm^2^. The OV for the generator was 7 mV at Δ*T* of 19 K and the output power was 2.1 μW at Δ*T* of 19 K. The thermoelectric generator had a voltage factor of 0.15 μV/mm^2^K and an output power of 2.33 pW/mm^2^K^2^. Oh [18] proposed a flexible thermoelectric generator for self-powered wearable electronics. The materials for the FTG were p-type NbSe_2_ and n-type WS_2_ nanosheet films. The flexible thermoelectric generator produced an output power of 38 nW at the Δ*T* of 60 K and the performance was stable after 100 bending cycles and 100 stretching cycles at a 50% strain. Kim [19] used an optimized composite film with tungsten disulfide nanosheets and single wall carbon nanotubes to fabricate a flexible thermoelectric generator on a rubber substrate with pre-strain. The FTG kept its performance after 10,000 stretching cycles at a 30% strain. These thermoelectric generators [13,14,15,16,17] are flexible and have more applications than non-flexible thermoelectric generators [9,10,11]. This study uses a low cost electroplating process that allows easy fabrication to manufacture a flexible thermoelectric generator on an epoxy substrate. The power factor for the FTG exceeds that for the FTG’s that were developed by Itoigawa [13], Lu [14], Ding [15], and Jo [17].

## 2. Design for the Thermoelectric Generator

**Figure 1:**
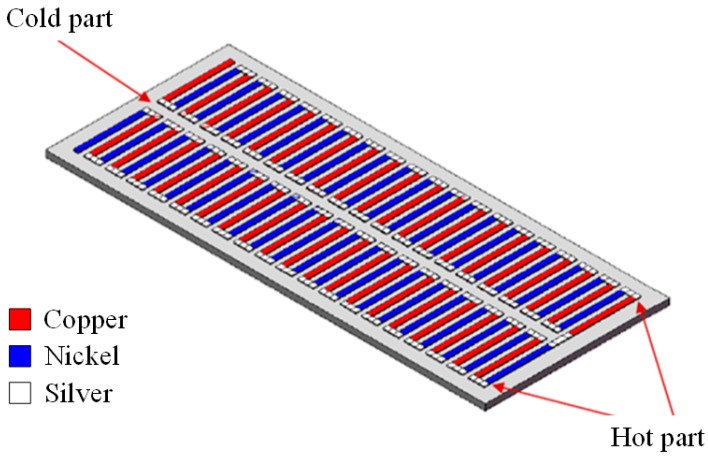
Schematic structure of the flexible thermoelectric generator.

**Figure 2:**
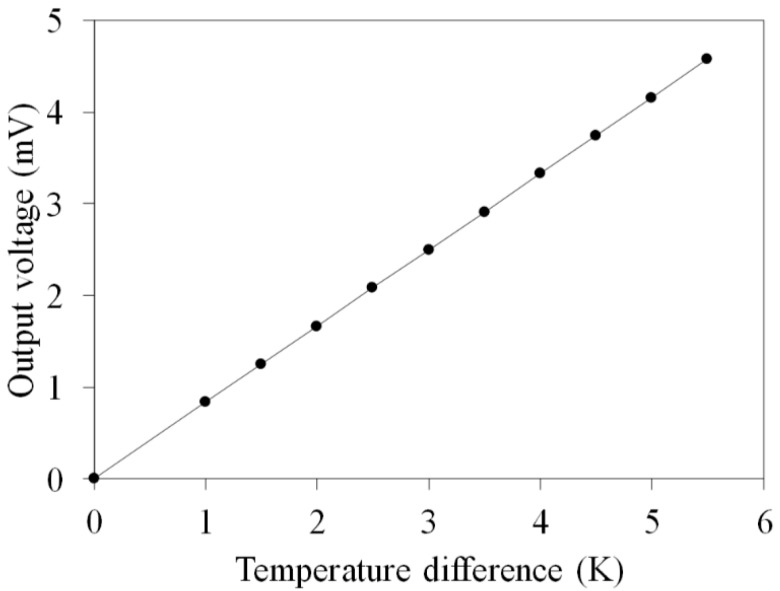
Simulated output voltage for the flexible thermoelectric generator.

Figure 1 shows the schematic structure of the flexible thermoelectric generator. The FTG is composed of 39 thermocouples in series. Each of the thermocouple is made of copper and nickel. The thermocouples have a hot part and a cold part. Each thermocouple is 7 mm long, 0.5 mm wide, and 0.1 mm thick. The area of the FTG is 46 × 17 mm^2^. The FTG uses the Seebeck effect to generate an output voltage if the hot and cold parts of the thermocouples have different temperatures. The output voltage for the FTG is given by [20],

(1) $$U_{0}=m\left(\alpha_{c}-\alpha_{n}\right)\Delta T$$

where *U*_0_ represents the OV for the FTG, *m* is the number of thermocouples in the FTG, *α_c_* is the Seebeck coefficient for copper, *α_n_* is the Seebeck coefficient for nickel and Δ*T* is temperature difference between the hot part and cold parts of the thermocouples. Equation (1) shows that that the OV for the FTG is proportional to the number of thermocouples (*m*), the difference in the Seebeck coefficients (*α_c_* − *α_n_*) for the thermocouple materials, and Δ*T* for the thermocouples [21]. If there is an increase in the three parameters, *m*, *α_c_* − *α_n_* and Δ*T*, the OV for the FTG increases. To increase the temperature difference between the hot and cold parts of thermocouples, the length of the thermocouples is extended to 7 mm. In the design, thermocouple materials are copper and nickel. The Seebeck coefficient of copper is 1.83 μV/K, and the Seebeck coefficient of nickel is −19.5 μV/K [22]. The thermocouple number of the FTG is 39. The values *m* = 39 and *α_c_* − *α_n_* = 21.33 μV/K are substituted into Equation (1), the relation between the OV and Δ*T* of the FTG is obtained. Figure 2 shows the simulated OV of the FTG. In this computation, the temperature difference of thermocouples changes from zero to 5.5 K. As shown in Figure 2, the change between the OV and Δ*T* was linear relation. The simulated OV of the FTG was 4.6 mV at Δ*T* of 5.5 K.

**Figure 3:**
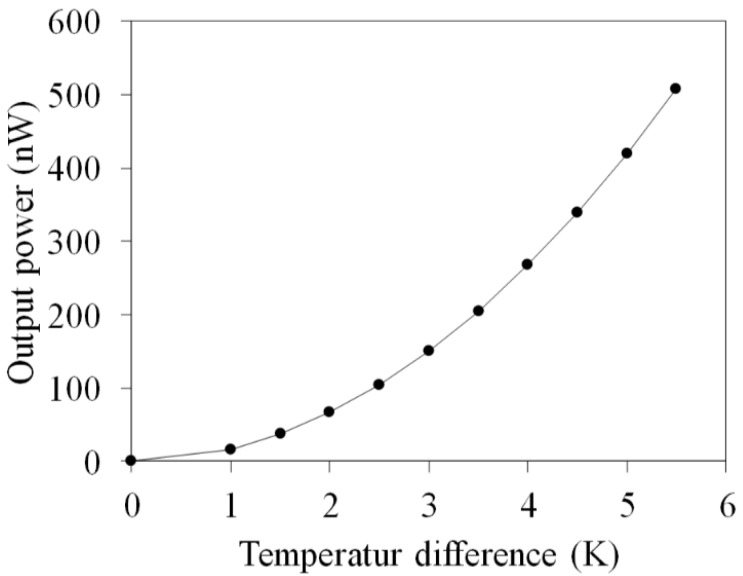
Simulated output power for a flexible thermoelectric generator.

The output power of the FTG depends on the internal resistance and the external load. When the internal resistance of the FTG equals its external load, the FTG has a maximum output power. The maximum output power of the FTG is given by [23],

(2) $$P_{max}\;=\;\frac{U_{0}{}^{2}}{{4R}_{g}}$$

where *P_max_* is the maximum output power for the FTG, *U*_0_ is the OV for the FTG, and *R_g_* is the internal resistance of the FTG. Equation (2) shows that the maximum output power of the FTG is proportional to the square of the OV and is inversely proportional to the internal resistance [24]. If the OV for the FTG is increased and the internal resistance is decreased, the maximum output power increases. The maximum output power for the FTG is calculated. The internal resistance of the FTG is assumed to be 10.3 Ω. The value *R_g_* = 10.3 Ω and the simulated OV in Figure 2 are substituted into Equation (2). The maximum output power for the FTG is calculated and the simulated results are shown in Figure 3. The temperature difference for the thermocouples changes from zero to 5.5 K. Figure 3 shows that the relationship between the output power and temperature difference is nonlinear. The simulated output power for the FTG is 508 nW at Δ*T* of 5.5 K.

## 3. Fabrication of the Thermoelectric Generator

**Figure 4:**
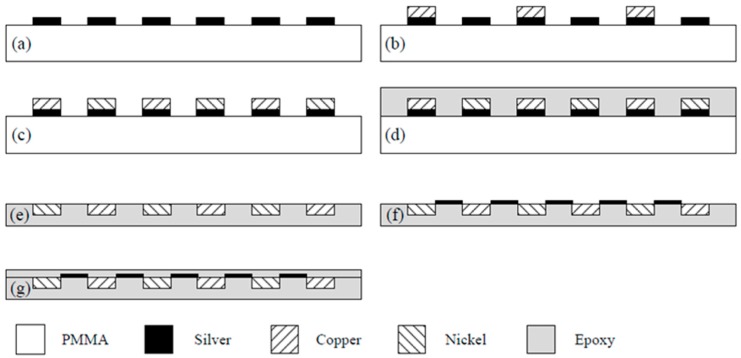
Process flow for a flexible thermoelectric generator: (**a**) Defining a silver seed layer; (**b**) electroplating copper; (**c**) electroplating nickel; (**d**) coating epoxy polymer; (**e**) etching polymethyl methacrylate substrate; (**f**) painting silver; (**g**) packaging.

The flexible thermoelectric generators were fabricated on an epoxy substrate using an electroplating technique. The structure of the FTG consisted of thermocouples in series. The materials for the thermocouples were copper and nickel. Figure 4 illustrates the process flow for the FTG.

**Figure 5:**
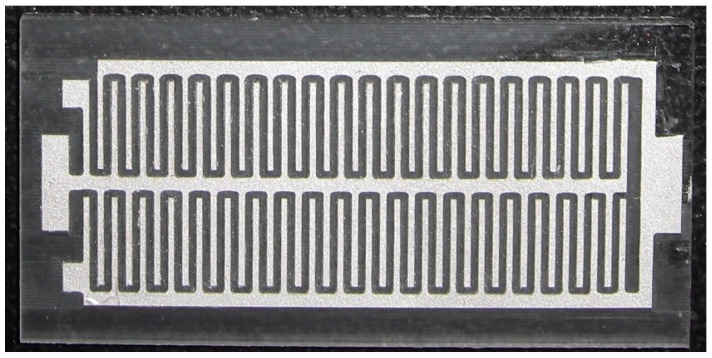
The pattern of the silver seed layer.

**Figure 6:**
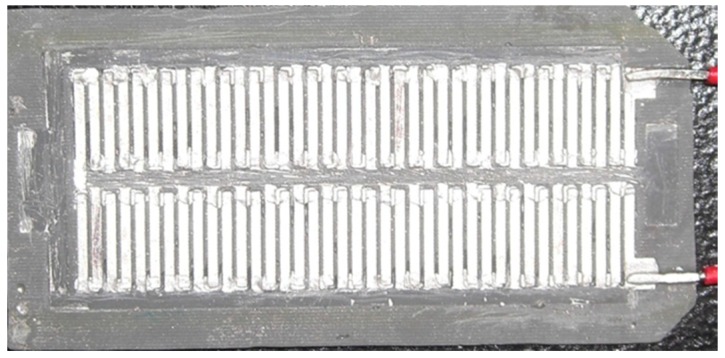
An image of the flexible thermoelectric generator.

**Figure 7:**
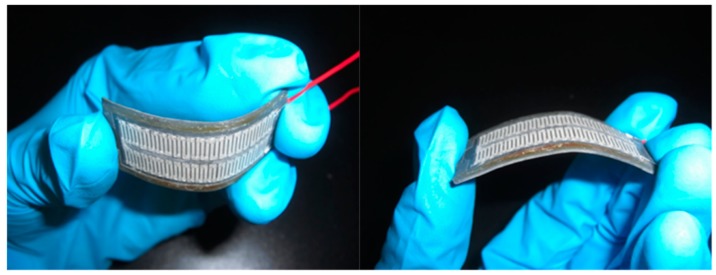
Demonstration of the flexibility of the flexible thermoelectric generator.

Figure 4a shows a seed layer of silver on the PMMA substrate. The silver seed layer was coated onto the PMMA substrate, and the silver layer was patterned using a computer numerical control (CNC) micro-milling machine. Figure 5 shows an image of the silver layer pattern that is defined by the CNC micro-milling machine. Figure 4b shows that a copper layer is deposited onto the silver layer. An electroplating process with CuSO_4_ solution and a current density of 4 A/dm^2^ for 45 min was utilized to deposit the copper layer onto the silver layer. The thickness of the copper layer was 100 μm. Figure 4c shows that a nickel layer is electroplated onto the silver layer. An electroplating process with NiSO_4_ solution and a current density of 4 A/dm^2^ for 45 min was employed to deposit the nickel layer onto the silver layer. The thickness of the nickel layer was 100 μm. Figure 4d shows that an epoxy is coated onto the PMMA substrate. Epoxy is used because the PMMA substrate is not flexible so epoxy is used to replace the PMMA substrate. Figure 4e shows etching the PMMA substrate. The FTG was immersed in an acetone solution for 30 min to etch the PMMA substrate and structure of the thermocouples was transferred to the epoxy substrate. Figure 4f shows that silver paint connects the nickel and the copper to form the thermocouples in series. Figure 4g shows that a thin epoxy layer is coated onto the FTG to protect the thermocouples. This layer prevents damage from dust and steam. Figure 6 shows an image of the flexible thermoelectric generator. Figure 7 shows that the FTG is flexible.

## 4. Results and Discussion

A heater, a cooler, a power supply, an infrared thermometer and a digital multimeter were used to test the OV for the FTG. The heater was placed at the hot part of the thermocouples and the cooler was placed at the cold part of the thermocouples. The power supply provided power to the heater and cooler. The heater acted as a heat source for the hot part of the thermocouples and the cooler acted as a heat sink for the cold part of the thermocouples. An infrared thermometer was used to measure the temperature difference between the hot and cold parts of the thermocouples. A digital multimeter recorded the OV for the FTG.

**Figure 8:**
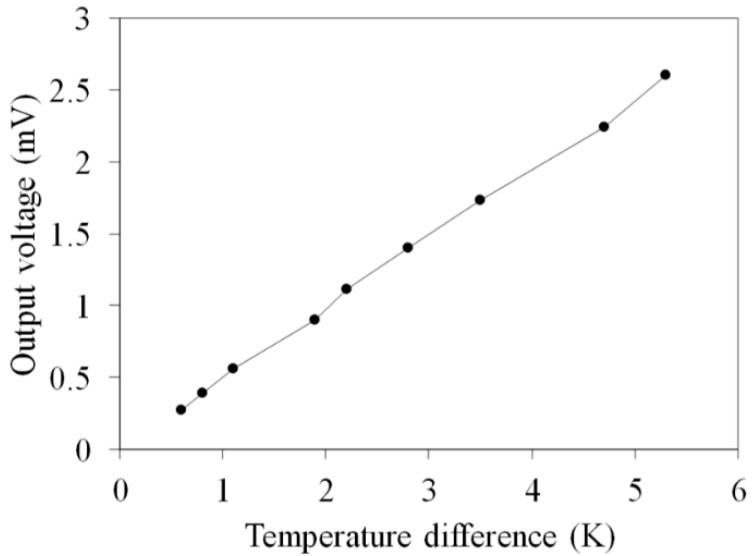
Measurements for the output voltage of the flexible thermoelectric generator.

**Figure 9:**
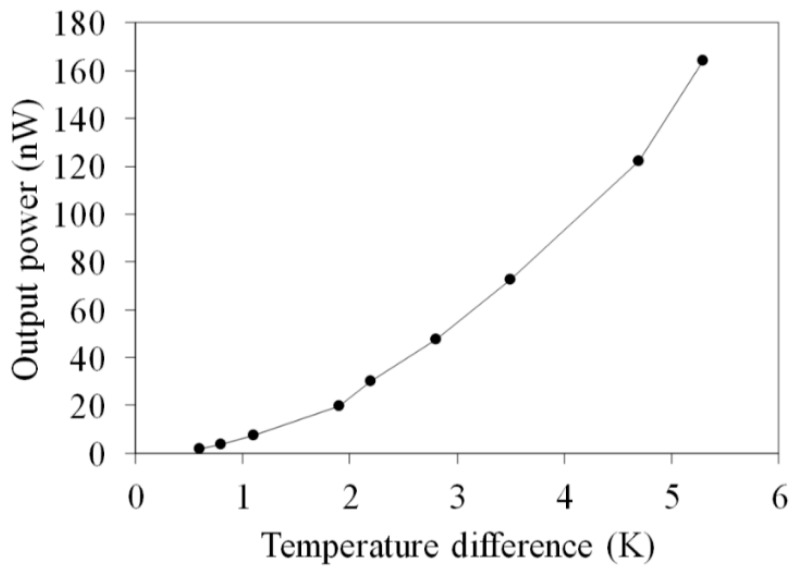
Measurements for the output power of the flexible thermoelectric generator.

Figure 8 shows the measured OV for the flexible thermoelectric generator. The temperature difference of the thermocouples in the FTG was varied between zero and 5.3 K. The results show that the OV for the FTG is 2.6 mV at Δ*T* of 5.3 K. The internal resistance was measured using a digital multimeter. The value was 10.3 Ω. Equation (2) is used to calculate the maximum output power for the FTG. The internal resistance *R_g_* = 10.3 Ω and the measured results for the FTG OV in Figure 8 are substituted into Equation (2) to calculate the maximum output power. Figure 9 shows the measured maximum output power for the flexible thermoelectric generator. The measured maximum output power for the FTG was 165 nW at Δ*T* of 5.3 K.

**Figure 10:**
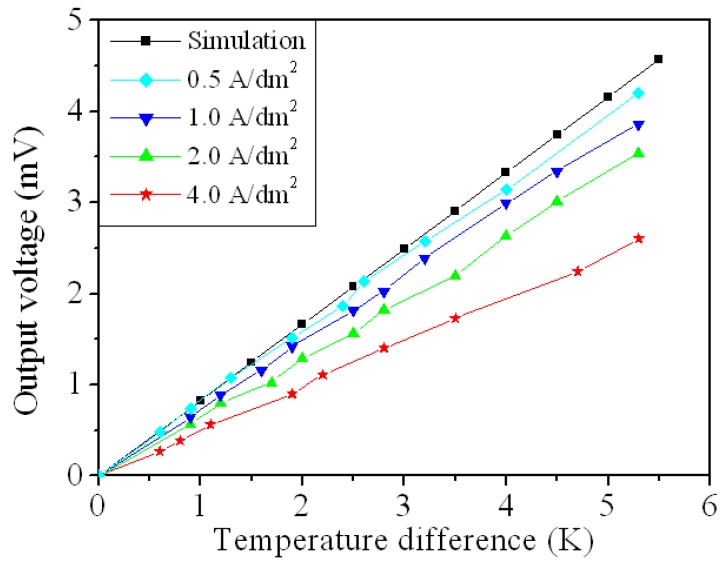
Output voltage of the flexible thermoelectric generator at different current density.

As shown in Figure 2 and Figure 8, the simulated OV for the FTG is 4.3 mV at Δ*T* of 5.3 K and the measured OV is 2.6 mV at Δ*T* of 5.3 K. A comparison of the simulated and measured results shows that the OV for the FTG has an error percentage of 40%. As shown in Figure 3 and Figure 9, this error results in a large difference between the simulated and the measured maximum output power. The thermoelectric properties of the copper and nickel in the thermocouples depend on the current density during the electroplating process. To reduce the error and improve the thermoelectric properties of the thermocouples, this study uses different values of current density to electroplate the copper and nickel for the FTG and measures the OV. Figure 10 shows the measured OV for the flexible thermoelectric generator for different electroplating current densities. Four current densities are used to electroplate the copper and nickel onto the FTG: 0.5 A/dm^2^, 1 A/dm^2^, 2 A/dm^2^ and 4 A/dm^2^. The results show that the OV for the FTG increases as the current density that is used for electroplating is decreased. As shown in Figure 10, the FTG produces the greatest OV at a current density of 0.5 A/dm^2^. The results (0.5 A/dm^2^) are in agreement with the simulation results. At a current density of 0.5 A/dm^2^, the FTG produces an OV of 4.2 mV for a value of Δ*T* of 5.3 K. The voltage factor for the FTG was 1 μV/mm^2^K. The values *U*_0_ = 4.2 mV, Δ*T* = 5.3 K and *m* = 39 are substituted into Equation (1) to evaluate the difference in the Seebeck coefficient for the thermocouple materials. The result shows that the difference in the Seebeck coefficients (*α_c_* − *α_n_*) for the thermocouple materials is 20.3 μV/K.

**Figure 11:**
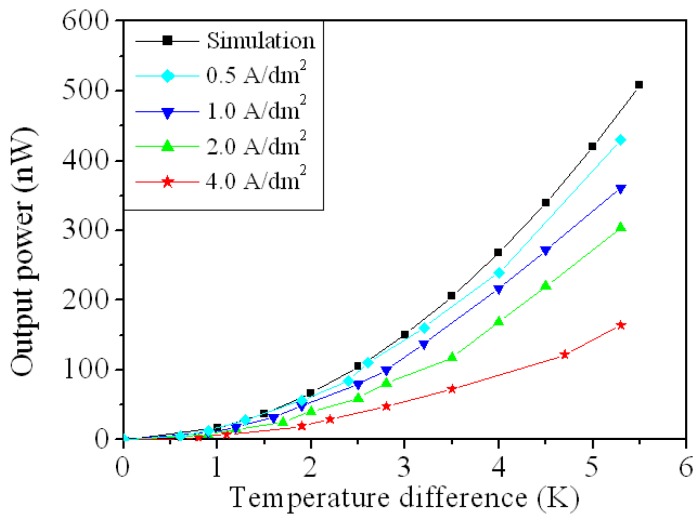
Output power of the flexible thermoelectric generator at different current density.

The results in Figure 10 are substituted into Equation (2) to calculate the maximum output power for the FTG. Figure 11 shows the maximum output power for the flexible thermoelectric generator for various values of current density for electroplating. The results show that the maximum output power for the FTG increases as the current density of electroplating is decreased. As shown in Figure 11, the FTG produces the greatest output power at a current density of 0.5 A/dm^2^. The results (0.5 A/dm^2^) are in agreement with the simulation results. At a current density of 0.5 A/dm^2^, the FTG produces an output power of 429 nW for a value of Δ*T* of 5.3 K. The power factor for the FTG was 19.5 pW/mm^2^K^2^.

**Figure 12:**
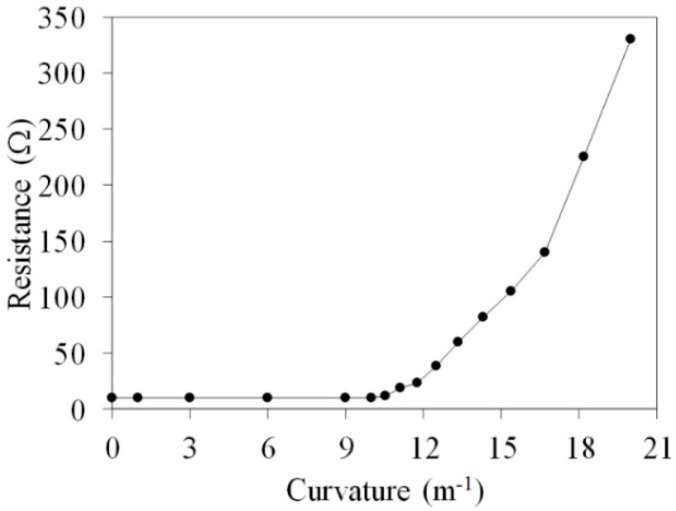
Relationship between resistance and curvature for the FTG.

The relationship between the curvature and resistance of the FTG is used to characterize the flexibility of the FTG. A digital multimeter was used to record the change in the resistance of the FTG when a force is applied to change its curvature. Figure 12 shows the relationship between the curvature and the resistance of the FTG. For a curvature of less than 10 m^−1^, the resistance of the FTG remains constant at 10.3 Ω. The resistance of the FTG increases from 10.3 to 330 Ω as the curvature of the FTG increase from 10 to 20 m^−1^. The limit of curvature for the FTG is 20 m^−1^, which corresponds to a radius of curvature that is 50 mm.

Table 1 lists the performance for various thermoelectric generators. These thermoelectric generators, which were fabricated by Itoigawa [13], Lu [14], Ding [15], Selvan [16], and Jo [17], were flexible. As shown in Table 1, the power factor for the FTG that was produced by Selvan [16] is 66 pW/mm^2^K^2^ which is the greatest power factor. The power factor for the FTG that was produced by Lu [14] is 0.064 pW/mm^2^K^2^, which is the lowest power factor. The power factor for the FTG that is produced by this study is 19.5 pW/mm^2^K^2^, which is a higher figure than that for the FTG’s that were produced by Itoigawa [13], Lu [14], Ding [15], and Jo [17].

The internal resistance of the thermoelectric generator that was fabricated by Peng [21] was 8 kΩ, and the internal resistance of the thermoelectric generator that was produced by Yang [23] was 8 kΩ. The thermoelectric generator that was produced by Kao [24] had an internal resistance of 2.45 kΩ. The internal resistance of the FTG that is produced by this study is 10.3 kΩ, which is a lower figure than those for the thermoelectric generators that were produced by Peng [21], Yang [23], and Kao [24].

## 5. Conclusions

A flexible thermoelectric generator was fabricated on an epoxy substrate using an electroplating process. The FTG contained 39 thermocouples in series. These thermocouples were composed of the thermoelectric materials, copper, and nickel. The electroplating process, which is low cost and allows easy processing, was used to deposit the copper and the nickel. The FTG is quite flexible, with a maximum curvature of 20 m^−1^. The experiments showed that the OV and maximum output power for the FTG were 4.2 mV and 429 nW, respectively, for a value of Δ*T* of 5.3 K. The FTG had a voltage factor of 1 μV/mm^2^K and a power factor of 19.5 pW/mm^2^K^2^.

## Figures and Tables

**Table 1:** Performances of various thermoelectric generators.

Authors	Flexibility	Voltage Factor (μV/mm^2^K)	Power Factor (pW/mm^2^K^2^)
Lee [9]	No	-	0.68
Phaga [10]	No	1.35	0.16
Big-Alabo [11]	No	-	35
Itoigawa [13]	Yes	2.43	0.64
Lu [14]	Yes	0.8	0.064
Ding [15]	Yes	1.5	1.2
Selvan [16]	Yes	-	66
Jo [17]	Yes	0.15	2.33
This work	Yes	1	19.5
